# Exploring the use of single session interventions in child and adolescent mental health services in England: a freedom of information-based study

**DOI:** 10.3389/fpsyg.2026.1636709

**Published:** 2026-02-04

**Authors:** Sophie Dallison, Grace Leigh, Chloe Payne-Cook, Philip John Archard, Windy Dryden, Maria Elizabeth Loades

**Affiliations:** 1 Department of Psychology, University of Bath, Bath, United Kingdom; 2 Department of Education and Training at Tavistock NHS, Tavistock and Portman NHS Foundation Trust, London, United Kingdom; 3 School of Criminology, University of Leicester, Leicester, United Kingdom; 4 Department of Social Therapeutic and Community Studies, Goldsmiths University of London, London, United Kingdom

**Keywords:** brief interventions, child and adolescent mental health, freedom of information, service delivery, single session, single session interventions, single session therapy

## Abstract

**Objective:**

We explored the provision of single-session interventions (SSIs) in public child and adolescent mental health service (CAMHS) provision, using freedom of information (FOI) requests to gather data from National Health Service (NHS) trusts.

**Method:**

FOI requests were sent to all NHS trusts in England with CAMHS in 2024, seeking information on SSI use, delivery, and implementation.

**Results:**

Responses from participating trusts indicated that most trusts did not use SSIs, with varied deployment of SSIs in those that provided them. Some trusts reported plans to expand SSI provision.

**Conclusion:**

Although SSIs are used in some CAMHS, variability in practice and reporting suggests a need for further research into decision-making and strategies for the implementation of SSIs in different localities.

## Highlights

- Single session interventions (SSIs) are intentionally designed to be delivered and completed in one session, without presuming a need for repeated attendance.
- Little is known about what SSIs are offered by child and adolescent mental health services (CAMHS) in England, to whom, and at what point in the care pathway.
- Despite their potential scalability and the proclivity of a substantial minority of service users to only attend once, our exploratory study found that most CAMHS providers are not offering SSIs.
- Where SSIs are offered, what is provided varies in delivery methods and implementation models/therapeutic modalities.

## Background

Single session interventions (SSIs), including single session therapies, are intentionally designed to be delivered and completed in one session, with more help available if required but not presumed (Dryden, 2020). The single session can be based on various therapeutic modalities, and offered to an individual or group, in person or virtually, and as self-help or practitioner facilitated. SSIs have been found to have a beneficial effect on a range of mental health difficulties, as evidenced in a recent umbrella review of 24 systematic reviews and meta-analyses (Schleider et al., 2025).

There are two main reasons why SSIs may be of particular interest and relevance to child and adolescent mental health services (CAMHS). Firstly, service pathways have traditionally tended to presume repeat attendance over several sessions during which benefits accrue, but prior research has found that almost half of children and young people (CYP) only attend once (Edbrooke-Childs et al., 2021). This echoes findings regarding service use across age groups (Schleider et al., 2025). Therefore, single session attendance is already the provision many are receiving, despite service pathways not being designed around a SSI model. Secondly, SSIs are potentially more scalable than multi-session alternatives (Schleider and Weisz, 2018; Schleider et al., 2020; Weisz et al., 2017), and could be lower-resource complements to multi-session care. In the current context in England, there is a considerable burden of mental health need (Newlove-Delgado et al., 2023). Owing to this level of need and because CAMHS provision is limited by the number of available trained professionals and funding constraints, waiting lists for treatment can be long (Hansen et al., 2021). SSIs could be sufficient to meet some CYP’s needs, and for others, could be a useful first step or an adjunct whilst waiting for further treatment.

Yet, little is known about what SSIs are offered by CAMHS in England, to whom, and at what point in the care pathway. We thus sought to map SSI provision via a preliminary, exploratory investigation. Specifically, we wanted to find out: 1. what SSIs are currently offered within CAMHS, 2. to whom, 3. when in the care pathway, and 4. which professional groups are involved in delivering them.

## Methods

### Sample

In August and September 2024, Freedom of Information (FOI) requests were sent to a total of 62 trusts in England. This sample of trusts was chosen due to their role as providers of CAMHS, and is inclusive of all such providers in England [as previously identified by and adapted from Valentine et al. (2023)].

### Data collection

In the UK, the Freedom of Information Act 2000 affords members of the public the statutory right to legally request data held by public authorities (Savage and Hyde, 2014), including regarding service provision in the NHS. Under the Act, authorities are required to state whether they hold the information requested and must disclose that information within set timescales, unless the data requested falls under certain exemptions designated in the Act. Despite being a typically overlooked data collection method within health and social care research, a FOI-based approach yields higher response rates than alternative methodologies, such as voluntary questionnaire-based surveys (Archard et al., 2023; Clifton-Sprigg et al., 2020). Prior studies have used FOI requests to obtain data relating to CAMHS and psychological therapy provision in the NHS (Crenna-Jennings and Hutchinson, 2020).

Given the aim of mapping SSI provision within CAMHS, a survey-based design was chosen. Qualitative and quantitative data was collected via a form sent to information governance professionals representing each trust. The form requested information regarding whether SSIs were delivered, where in service provision they were implemented, intervention modalities, and occupational groups involved in intervention delivery.

Following an introductory email sent to each trust’s designated FOI contact (see Supplementary material), we piloted the FOI request with two NHS trusts in August 2024. FOI representatives responded to and provided feedback on the requests. Subsequently, after minor changes to wording, we sent the requests by email to the remaining 60 trusts. It was not possible to determine how the respondents, as information governance professionals, gathered information for responses, raising some potential for discrepancies between what administrative data indicates and actual clinical practice.

### Data analysis

The final dataset comprised data from 42 trusts (67.7% response rate). Quantitative data were entered into a Microsoft Excel file and analysed descriptively, with counts and percentages reported. Free text qualitative responses were analysed by two members of the research team using inductive content analysis (Vears and Gillam, 2022). Given the exploratory nature of this study and that qualitative information provided was variable in scope and quality, inductive content analysis was deemed the most appropriate analytic method.

Two team members undertook the content analysis, first by reading and familiarising themselves with the data, then coding individual responses provided by trust representatives to consider general commonalities and differences. This coded data was then revisited for further, fine grained coding (on a line-by-line basis) to explore, in greater depth, how information was related in responses and specific details provided.

### Ethical considerations

As relevant data were obtained via FOI requests, no formal institutional ethical review was required (Walby and Luscombe, 2018). Only aggregate institutional data were collected, with all responses anonymised.

## Results

Of the 42 trusts who responded to the FOI request, 12 (28.6%) of whom indicated that they provide SSIs to CYP, with 27 (64.3%) not providing SSIs. Three (7.1%) responses did not answer this question. Thus, what follows is based on the responses from the 12 trusts who provide SSIs to CYP.

### Target populations

All 12 trusts providing CAMHS who offer SSIs reported delivering them for CYP. Six trusts provided SSIs to parents/carers and one trust to professionals.

### Professional groups providing SSIs

The 12 trusts offering SSIs typically selected multiple options from the list of occupational groups we provided establishing that different groups are involved in SSI delivery, with between one and ten occupational groups depending on the trust (*M* = 4.6; *Mdn* = 5). Where specified, the most commonly involved professional groups were mental health nurses (9 trusts), practitioner psychologists (8 trusts), and mental health practitioners (including education mental health practitioners and child wellbeing practitioners) (7 trusts). SSIs were also delivered by social workers (6 trusts), trainee psychologists/clinical associate psychologists (4 trusts), assistant psychologists (3 trusts), systemic psychotherapists/family therapists (3 trusts), occupational therapists (3 trusts), and counsellors/psychotherapists (3 trusts) [with one trust specifying psychoanalytic psychotherapists and another, cognitive behavioural therapists/staff trained in cognitive behavioural therapy (CBT)], as well as consultant psychiatrists (2 trusts) and psychiatry registrars (2 trusts). One trust reported that support workers were involved in delivering SSIs. See also Table 1.

**Table 1:** Principal occupational groups involved in SSI delivery.

Occupational group	Number of trusts
Mental health nurse	9
Practitioner psychologist	8
Mental health practitioner (including education mental health practitioners and child wellbeing practitioners)	7
Social worker	6
Trainee psychologist/Clinical associate psychologist	4

### Where in the care pathway SSIs are offered

Ten of the 12 (83.3%) trusts reported providing SSIs as a first step to accessing help and nine (75%) as an additional source of help alongside other provisions such as medication or psychological therapy beyond a single session. Seven (63.6%) trusts provided SSIs as a crisis intervention.

In additional information regarding where SSIs were offered in care pathways and service provision, this included in Mental Health in Schools Teams as a psychoeducation workshop, via a drop-in service, as part of discharge pathway pilot, as an early/preventative intervention, and as part of general hospital liaison provision. One trust described how SSIs were being embedded into Single Point of Access provision, in a crisis team and in a newly introduced discharge pathway. Two trusts referred to primary mental health care provision, one in the form of mental health practitioners in primary care networks providing SSIs to CYP presenting to primary care with mental health needs.

The four categories of presenting problem/foci we identified for which SSIs are offered based on information obtained from six trusts (see Table 2) indicated that SSIs were being used for various presenting problems/foci. The most commonly identified category was psychological wellbeing, including anxiety, low mood and stress, followed by sleep difficulties, then social and relational problems, with some focus on school related concerns, for instance relating to bullying and school transitions.

**Table 2:** Foci for SSIs.

Category	Example responses (from trust responses)
Psychological wellbeing (6 trusts)	Anxiety and social anxiety, low mood, stress, emotional dysregulation
Sleep (4 trusts)	Sleep difficulties, sleep hygiene
Social and relational problems (3 trusts)	Friendship difficulties, social media and wellbeing, anti-bullying, school transitions
Neurodivergence (2 trusts)	Autism, ADHD

Variability was captured in SSI type in format, context, and modality. Of the six trusts that provided information regarding format and context, all reported that SSIs were delivered in workshops in comparison to the one trust that reported them as being provided through drop-in sessions. Ten trusts provided information regarding SSI modalities, with psychoeducation (9 trusts) and CBT-informed or CBT-based interventions (4 trusts) being most commonly used. One trust response noted staff training in the Dryden model of single session therapy and the use of principles from dialectical behavioural therapy, another, the Bouverie Centre Model of ‘single session thinking’. When exploring relationships between presenting problems/foci and SSI format and modality, psychoeducation, typically through a workshop format, was more commonly used for anxiety, sleep difficulties, and low mood. The categories of CBT-informed and psychoeducation modalities were, in some instances, overlapping, as workshops for CYP and parents/carers could be based on CBT frameworks.

### Additional information provided by trusts

In addition to the information we specifically requested, three trusts provided additional information regarding service-wide implementation of SSI frameworks and models. This information referenced plans for future development of SSI implementation, notably in commissioning workforce training and extending a ‘single session thinking’ approach in routine care delivery.

## Discussion

As a preliminary, exploratory investigation using a FOI-based approach to gather data, we mapped SSI provision across NHS trusts providing CAMHS in England and found that most trusts are not using SSIs within their service provision. In the trusts that did report using SSIs, what was being offered, for whom, by whom and at what point in the care pathway was varied.

Introducing SSIs as part of their service provision could be important for NHS trusts and commissioners to consider, given the advantages SSIs offer in terms of potential efficiency and scalability, and the reality that almost half of recipients of CAMHS-based support only attend once (Edbrooke-Childs et al., 2021). Internationally, the evidence base for SSIs is strong (Schleider et al., 2025), including for youth mental health specifically (Schleider and Weisz, 2017; Ball et al., 2024; Hatoum et al., 2025). SSIs also align well with service delivery frameworks which are already in widespread use in the NHS and in CAMHS specifically, for example, the influential THRIVE framework for system change in mental health services for children, young people and their families, which positions single session delivery as part of an integrated, needs led, and person-centred approach to care delivery (Wolpert et al., 2019). Specifically, THRIVE foregrounds effective prevention and promotion strategies, and SSIs can provide goal-based and needs-led help, consistent with this framework. To support the introduction of SSIs, further research is needed to explore commissioner and service leader decision-making regarding the use and funding of SSIs. Although our findings suggests that SSIs may not be consistently or optimally deployed in CAMHS in England, local decision-making may involve complex considerations about what is both effective and economical.

Where SSIs are offered, there may be several reasons for different areas offering different single session approaches and provision. The needs of the population may differ in different geographical areas; for example, populations in more rural areas may be less able to regularly attend clinic based locations which tend to be in urban centres (Schleider et al., 2020). Populations in more deprived areas may also be disproportionately vulnerable to a high level of mental health need (Díaz et al., 2022) and therefore demand may be higher, driving the need for adaptations in service delivery approaches (Holding et al., 2022) like SSIs. However, differences in provision may also be indicative of place-based healthcare inequalities (Redhead and Lynch, 2024). Concerningly, large scale data collected anonymously through schools in England indicates that students who had experienced adversity are more likely both to have previously accessed mental health support and yet also to experience this support as not meeting their needs (Soneson et al., 2024), which suggests a widespread need to better match what is provided to the needs of those who are most vulnerable to experiencing mental health problems.

Self-administered SSIs, which are the most scalable form of SSIs, have small but significant effects on reducing anxiety and depression symptoms in children and young people (Ball et al., 2024). This provision format could be a useful way to expand CAMHS pathways to including more accessible intervention options without needing to increase the therapist workforce and with minimal cost. Pilot studies in the UK context have found that this SSI format is promising, including in the NHS (Ching et al., 2023) and the community (Ball et al., 2025; Perkins et al., 2021; Loades et al., 2025), although longer term effectiveness is yet to be investigated. Further research is also needed to establish how such interventions could best be integrated into routine care, including by investigating the factors influencing their adoption, and their impact on clinical outcomes. This would lead to a clearer picture of when, where and how to implement SSIs to enhance CAMHS provision.

### Strengths and limitations

A strength of this study is the use of a FOI-based approach, as a novel method for gathering data across NHS trusts in England, offering a means of collecting data to map provision across multiple areas (Archard et al., 2023). However, responses to the FOI request were provided by information governance professionals, therefore there is the possibility that responses do not fully capture care delivered, depending on how information was gathered through dialogue between information governance, clinical leaders and frontline professionals. Additionally, only two-thirds of trusts we contacted responded, with a quarter of these offering SSIs, placing some limitations on the completeness and generalisability of the data.

## Conclusion

Despite their potential scalability and the proclivity of a substantial minority of users to only attend once, many CAMHS in England do not appear to be using SSIs, thus further research is needed to understand the reasons for this. Among those that do use SSIs, there appears to be variability in target populations, delivery methods, and implementation. SSIs could be deployed by CAMHS providers to support timely, accessible mental health care for children and young people, and further work to examine their effectiveness and implementation is merited.

## Data Availability

The original contributions presented in the study are included in the article/Supplementary material, further inquiries can be directed to the corresponding author/s.
