# Supplementary material for: Exploring the use of single session interventions in child and adolescent mental health services in England: a freedom of information-based study
Source: Front Psychol. 2026 Feb 4;17:1636709. doi: 10.3389/fpsyg.2026.1636709 (PMC12913554; doi:10.3389/fpsyg.2026.1636709)
Supplement: Supplementary file 1 [file Supplementary_file_1.docx]

Dear FOI officer,

Under the Freedom of Information Act (2000) we would like to make a request for information relating to the provision of Single Session Interventions (SSIs) within Child and Adolescent Mental Health Services (CAMHS) within Dorset Health Care University Foundation Trust  from the financial year 2023-24. You can complete this in a body email reply or by filling in the attached word document version and sending it on return email as an attachment.

**Name of trust:**Dorset Health Care University Foundation Trust

A **Single Session Intervention (SSIs)** is a planned one-time intervention that is designed to provide individuals with immediate support and guidance for a specific issue or problem. That is, it is therapeutic in intent, and delivered as a standalone intervention, without presuming follow up or repeat use. It includes ‘single session therapy’, provided by a therapist from any therapeutic modality, and also self-help interventions designed intentionally to be used just once. Note that a one-off assessment (without providing guidance or therapeutic support) is not considered to be a single session intervention.

Our FOI request consists of 4 questions, pertaining to the financial year 2023-24:

1. Please mark X in the appropriate box:

|  | Yes | No |
| --- | --- | --- |
| Did your trust provide SSIs to Children and Young People (CYP)? |  |  |

1. If yes, what are these? (please give as much detail as possible about these, including whether these are individual/group, aimed at a specific type of disorder or age group, what therapeutic modality/model these are based on)

|  |
| --- |

1. Who delivered these interventions? (please mark X for all that apply)

| Practitioner Psychologists (incl. Clinical Psychologists, Forensic Psychologists, Counselling Psychologists, etc) |  |
| --- | --- |
| Assistant Psychologists |  |
| Trainee Psychologists/Clinical Associate Psychologists |  |
| Consultant Psychiatrists |  |
| Trainee Psychiatrists/Registrars |  |
| Support Workers |  |
| Mental Health Nurses |  |
| Mental Health Practitioners (including Education Mental Health Practitioners, Child Wellbeing Practitioners) |  |
| Family therapists |  |
| Occupational therapists |  |
| Social Workers |  |
| Psychoanalytic Psychotherapists |  |
| Other (please explain)s |  |

1. At what point(s) in the care pathway are SSIs are offered to CYP/families? (Please mark X to all that apply)

| Whilst waiting |  |
| --- | --- |
| As an additional source of help to users offered other therapeutic provision like medication or psychological therapy |  |
| As a first step to accessing help |  |
| As a crisis intervention |  |
| Other (please explain) |  |
